# Infrared Reflectance Analysis of Epitaxial n-Type Doped GaN Layers Grown on Sapphire

**DOI:** 10.1186/s11671-017-2171-0

**Published:** 2017-06-08

**Authors:** Bogdan I. Tsykaniuk, Andrii S. Nikolenko, Viktor V. Strelchuk, Viktor M. Naseka, Yuriy I. Mazur, Morgan E. Ware, Eric A. DeCuir, Bogdan Sadovyi, Jan L. Weyher, Rafal Jakiela, Gregory J. Salamo, Alexander E. Belyaev

**Affiliations:** 1 V. Lashkaryov Institute of Semiconductor Physics, National Academy of Sciences of Ukraine, Pr. Nauky 41, Kiev, 03680 Ukraine; 2 Institute for Nanoscience and Engineering, University of Arkansas, West Dickson 731, Fayetteville, AR 72701 USA; 3 Institute of High Pressure Physics, Polish Academy of Sciences, Sokolowska str. 29/37, 01-142 Warsaw, Poland; 4 Institute of Physics, Polish Academy of Sciences, Al. Lotnikow 32/46, PL-02-668 Warsaw, Poland

**Keywords:** Gallium nitride, Heterostructure, IR reflectance, Transfer matrix method, Carrier concentration, Mobility, Photo-etching, SIMS

## Abstract

Infrared (IR) reflectance spectroscopy is applied to study Si-doped multilayer n^+^/n_0_/n^+^-GaN structure grown on GaN buffer with GaN-template/sapphire substrate. Analysis of the investigated structure by photo-etching, SEM, and SIMS methods showed the existence of the additional layer with the drastic difference in Si and O doping levels and located between the epitaxial GaN buffer and template. Simulation of the experimental reflectivity spectra was performed in a wide frequency range. It is shown that the modeling of IR reflectance spectrum using 2 × 2 transfer matrix method and including into analysis the additional layer make it possible to obtain the best fitting of the experimental spectrum, which follows in the evaluation of GaN layer thicknesses which are in good agreement with the SEM and SIMS data. Spectral dependence of plasmon-LO-phonon coupled modes for each GaN layer is obtained from the spectral dependence of dielectric of Si doping impurity, which is attributed to compensation effects by the acceptor states.

## Background

In recent years, there has been high interest in III-nitride materials, in particular to GaN [1, 2]. Due to the breakthrough in the growth techniques, epitaxial GaN films have found wide application in optoelectronic devices such as blue and ultraviolet light emitting diodes (LEDs) [3], lasers [4], and microelectronic devices, e. g., high-power and high-frequency field effect transistors [5, 6]. Concentration and mobility of free carriers are the key parameters which determine the performance of the device in applications. Hall measurement of concentration and mobility of free carriers in multilayer GaN-based device structures is not trivial and time-consuming technological task which needs ohmic contacts attached to each measuring layer and dedicated measuring procedures.

Fourier transform infrared (IR) reflectance spectroscopy and Raman spectroscopy are contactless and non-destructive methods which allow for studying not only the phonon vibrations but also for characterizing the carrier properties [7]. However, the known problem of confocal micro-Raman spectroscopy is a deterioration in depth spatial resolution due to the refraction of light [8]. It was shown in ref. [6] that at depth scanning of multilayer GaN structure with an excitation wavelength of 488.0 nm, the depth resolution makes only about 1.8 μm while the lateral resolution is about 210 nm. IR spectroscopy overcomes this problem due to high sensitivity to layer thickness due to interference effects and impact of the dispersion of refractive index in a wide spectral range.

IR reflectance spectra of thin GaN films were investigated as far back as in 1973 by A.S. Baker [9], but spatial inhomogeneity and overall low structural quality of such films significantly limited the practical application of the obtained results. Nevertheless, a possibility to determine parameters of optical phonons and free carriers’ absorption in thin films of GaN was demonstrated. The detailed studies of longitudinal optical phonon*–*plasmon coupled (LOPC) modes in bulk GaN were performed by Perlin et al. [10] using Raman spectroscopy and by Shubert et al. [11] using IR ellipsometry. Effect of different substrates on the optical properties of cubic and wurtzite GaN films also has been studied in details [12, 13]. Considering the lack of native GaN substrates, it was shown that using sapphire substrates for epitaxial growth of GaN film is optimal for exploiting in devices which operate at high temperatures. IR reflection spectroscopy studies of hexagonal sapphire [14] showed a complex spectrum, the shape of which strongly depends on the polarization and the angle of incidence. This greatly complicates measurements and determination of the spectral characteristics of phonon modes and properties of free carriers in thin GaN film grown on sapphire substrates.

Thus, proper selection of spectral analysis algorithm and the form of dielectric function are of great importance for the analysis of the IR reflectance spectra of multilayer GaN-on-sapphire structures [15–17]. This paper shows a possibility of application of IR reflectance spectroscopy and 2 × 2 transfer matrix method for the analysis of planar GaN-based multilayer structures with non-uniform depth and doping profiles, which in practice can be different type of semiconductor III-nitride-based device structures with vertical design, such as light-emitting and rectifying diodes, Gunn diodes, high electron mobility transistors (HEMTs), etc.

## Methods

### Experimental

**Fig. 1:**
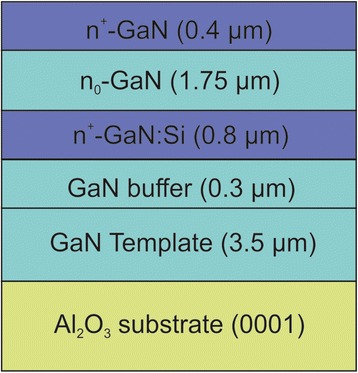
Schematic of the investigated n^+^/n_0_/n^+^-GaN structure grown on GaN-template/(0001) sapphire substrate

The investigated n^+^/n_0_/n^+^-GaN structures were grown on MOCVD GaN templates on Al_2_O_3_ (0001) substrates at a temperature of 800 °C by plasma-assisted molecular-beam epitaxy using an N_2_ flow rate of 0.5 sccm and an RF plasma power of 350 W (Fig. 1). This results in a growth rate of ∼ 0.27 ML s^−1^. First, a 0.3-μm-thick GaN buffer was grown on MOCVD GaN template. A 0.8-μm-thick Si-doped GaN layer was followed by a 1.75-μm-thick undoped GaN layer and a 0.4-μm-thick Si-doped GaN layer (Fig. 1). The nominal Si doping concentration of the n^+^-GaN layers was ∼ 10^19^ cm^−3^.

In order to examine the areas of different carrier concentration, the cleaved edge of the investigated structure was examined by the photo-etching method in an electroless configuration using K_2_S_2_O_8_–KOH aqueous solution (KSO-D etching system) [18]. This method allows revealing the areas of different carrier concentration and visualizing the relative carrier concentration differences by measuring the etch rate using surface profiling [19, 20]. Cross-section of the investigated sample was photo-etched for 3 min. Afterwards, samples were examined by scanning electron microscopy (SEM).

Secondary ion mass spectroscopy (SIMS) studies of samples were performed on a CAMECA IMS6F system using a cesium (Cs^+^) primary beam, with the current kept at 400 nA in order to find the profile of the impurities concentration. The size of the raster was about 50 × 50 μm^2^, and the secondary ions were collected from a central region of 30 μm in a diameter. For H, C, O, and Si, the concentrations were derived from H–, O–, C–, Si– species, respectively, and the matrix signal Ga– was taken as the reference.

The infrared reflectance spectroscopy measurements in the spectral range of 300–4000 cm^−1^ with the spectral resolution of 1 cm^−1^ were performed at room temperature using Bruker Vertex 70 V FTIR spectrometer equipped with Globar source and a deuterated triglycine sulfate (DLaTGS) detector with polyethylene window. The angle of incidence was 11°. *S*-polarized spectra were measured using KRS-5 polarizer. The reflectance spectrum of a gold mirror was used as a reference.

### Description of the Optical Analysis Model

The reflectance of layers/substrate system was calculated using the 2 × 2 transfer matrix method [17, 21] in which an arbitrary number of layers can be included and interference effects within the films are automatically considered. 2 × 2 transfer matrix method for isotropic layered systems allows for an independent calculation of *s*- and *p*-polarized reflection and transmittance spectra in the case of layered systems consisting of homogeneous biaxial or uniaxial isotropic slabs having their c-axis aligned with the z-axis of laboratory coordinates. In this case, 2 × 2 layered system transfer matrix can be represented in the following view [21]:

1 $$ {\left(\begin{array}{c}\hfill {E}_0^{+}\hfill \\ {}\hfill {E}_0^{-}\hfill \end{array}\right)}_{s/p}=\frac{1}{t_{0,1}^{s/p}}\left(\begin{array}{cc}\hfill 1\hfill & \hfill -{r}_{1,0}^{s/p}\hfill \\ {}\hfill {r}_{1,0}^{s/p}\hfill & \hfill 1\hfill \end{array}\right){\displaystyle \prod_{l=1}^N{T}_{l/\left(l+1\right)}^{s/p}{\left(\begin{array}{c}\hfill {E^{\prime}}_{N+1}^{+}\hfill \\ {}\hfill {E^{\prime}}_{N+1}^{-}\hfill \end{array}\right)}_{s/p}}={\left(\begin{array}{cc}\hfill {T}_{11}\hfill & \hfill {T}_{12}\hfill \\ {}\hfill {T}_{21}\hfill & \hfill {T}_{22}\hfill \end{array}\right)}_{s/p}{\left(\begin{array}{c}\hfill {E^{\prime}}_{N+1}^{+}\hfill \\ {}\hfill {E^{\prime}}_{N+1}^{-}\hfill \end{array}\right)}_{s/p}. $$

Asterisks in the top indexes of field amplitude in the exit medium are used in Eq. (1) to account for the values of electric field components just at the right side of the *N*/*N* + 1 interface.

The $2\times 2\kern0.24em {T}_{l,\left(l+1\right)}^{s/p}$ transfer-matrix accounts for the propagation of plane waves from the *l*-th layer, multiple reflections within this layer, and influence of *l/(l + 1)* interface. Such matrix can be determined as [17]:

2 $$ {T}_{l/\left(l+1\right)}^{s/p}=\frac{1}{t_{l/\left(l+1\right)}^{s/p}}\left(\begin{array}{cc}\hfill \exp \left(i{\delta}_l^{s/p}\right)\hfill & \hfill -{r}_{l+1,l}^{s/p} \exp \left(i{\delta}_l^{s/p}\right)\hfill \\ {}\hfill {r}_{l,l+1}^{s/p} \exp \left(-i{\delta}_l^{s/p}\right)\hfill & \hfill \exp \left(-i{\delta}_l^{s/p}\right)\hfill \end{array}\right), $$

where ${r}_{l,l+1}^{s/p}$ and ${t}_{l,l+1}^{s/p}$ denote partial reflection and transmission coefficients for *l/(l + 1)* interface, ${\delta}_l^{s/p}$ is the phase shift, imposed to light after propagation by the *l-*th layer for *s*- and *p*-polarized light.

Phase shift for *s*- and *p*-polarized light after passing through the *l-*th layer can be determined as [17]:

3 $$ {\delta}_l^{s/p}=\frac{2\pi {d}_l}{\lambda }{n}_{l,s/p} \cos {\theta}_{l,s/p}=\frac{2\pi {d}_l}{\lambda }{n}_l\sqrt{1-{\left(\frac{1}{n_{l,s/p}} \sin \theta \right)}^2}, $$

where *n*_*l*_ is the complex refractive index for the *l-*th layer, *d*_*l*_ is the thickness of the *l-*th layer, and *θ* is the angle of incidence.

Partial reflection and transmission coefficients for the *s*- and *p*-polarizations can be calculated using Fresnel equations. For example, partial reflection and transmission coefficients for the *s*-polarization have the following form [21]:

4 $$ \begin{array}{l}{r}_{l,l+1}^s=\frac{n_{ls} \cos {\theta}_{ls}-{n}_{\left(l+1\right)s} \cos {\theta}_{\left(l+1\right)s}}{n_{ls} \cos {\theta}_{ls}-{n}_{\left(l+1\right)s} \cos {\theta}_{\left(l+1\right)s}}\\ {}{t}_{l,l+1}^s=\frac{2{n}_{ls} \cos {\theta}_{ls}}{n_{ls} \cos {\theta}_{ls}+{n}_{\left(l+1\right)s} \cos {\theta}_{\left(l+1\right)s}}\end{array} $$

The complex reflectance ratios of the multilayer stack can be thus obtained by substituting the partial reflection and transmission coefficients for the *N + 1* interface (Eqs. (4)) in Eq. (1) and phase shifts of all the *N* layers (Eq. (3)):

$$ {R}_{s/p}={\left|{r}_{0,N+1}^{s/p}\right|}^2={\left|\frac{T_{21}}{T_{11}}\right|}^2. $$

### IR Dielectric Function Model

Refractive index depends on the complex dielectric function *ε*(*ω*), which can be written as:

5 $$ \varepsilon \left(\omega \right)={\varepsilon}^{\mathrm{lat}}\left(\omega \right)+{\varepsilon}^{\mathrm{fc}}\left(\omega \right). $$

The first term corresponds to contribution from lattice mode dispersion, and the second one to free carrier excitations.

The contribution of lattice modes to the IR response *ε* ^lat^(*ω*) at phonon energy *ℏω* can be described using a factorized model with Lorentzian broadening [22]:

6 $$ {\varepsilon}^{\mathrm{lat}}\left(\omega \right)={\varepsilon}_{\infty }{\displaystyle \prod_{k=1}^M\frac{\omega_{\mathrm{LO}k}^2-{\omega}^2-i\omega {\gamma}_{\mathrm{LO}k}}{\omega_{\mathrm{TO}k}^2-{\omega}^2-i\omega {\gamma}_{\mathrm{TO}k}}}, $$

where *M* is the number of infrared-active polar phonon modes for *s*- or *p*-polarizations to the *c-*axis; ω_LOk_ and ω_TOk_ are the frequency (cm^−1^) of the *k-*th LO and TO phonon; γ_LOk_ and γ_TOk_ are their damping constants (cm^−1^). For GaN the parameters ω_LOk_ and ω_TOk_ account for the *E*_1_(LO), *A*_1_(LO) and *E*_1_(TO), and *A*_1_(TO) vibrational modes [23].

The contribution of the free carrier species *ε* ^fc^(*ω*) to the dielectric function can be described using classical Drude approximation [15]:

7 $$ {\varepsilon}^{\mathrm{fc}}\left(\omega \right)=-{\varepsilon}_{\infty}\frac{\omega_p^2}{\omega \left(\omega +i{\gamma}_p\right)}, $$

with

8 $$ {\omega}_p={\left(\frac{N{e}^2}{\varepsilon_{\infty }{\varepsilon}_0{m}^{\ast }}\right)}^{1/2} $$

9 $$ {\gamma}_p=\frac{e}{m^{\ast}\mu } $$

The screened plasma frequencies *ω*_*p*_ (Eq. (8)) depend on the free carrier concentration *N*, high-frequency dielectric permittivity *ε*_∞_, and the effective mass *m* ^∗^ of the free carriers (*ε*_0_is the vacuum permittivity and *e* is the electrical unity charge). The plasmon damping parameter *γ*_*p*_ depends on the optical carrier mobility *μ* (Eq. (9)) [24].

Parameters of ω_LO_ and LOPC modes can be determined from the imaginary part of the energy loss function—$\mathrm{I}\mathrm{m}\left(-\frac{1}{\varepsilon \left(\omega \right)}\right)$ [7], where *ε*(*ω*) is the complex dielectric function, obtained from Eq. (5).

## Results and Discussion

**Fig. 2:**
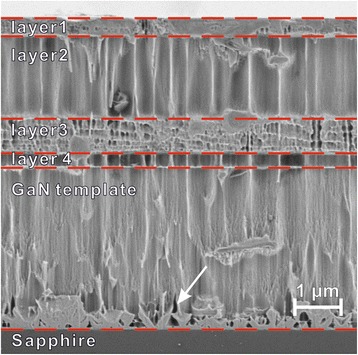
SEM image of cross-section of the investigated n^+^/n_0_/n^+^-GaN structure. The irregular pattern of vertical lines was formed during cleaving (i.e., before photo-etching) and is characteristic for the non-polished cleavages of Al_2_O_3_/GaN hetero-structures. Rough pyramidal layer (*pinholes*) at the sapphire/GaN template indicated by the *arrow* was revealed by photo-etching

SEM image (Fig. 2) shows the photo-etched cross section of n^+^/n_0_/n^+^-GaN structure grown on GaN-buffer/GaN-template/sapphire substrate, where six distinct layers are clearly visible, which are five GaN layers with different carrier concentration and sapphire substrate. It should be noted that the overall thickness of the investigated GaN structure as measured by SEM agrees with the technological one, and observed GaN layers according to Fig. 1 can be tentatively attributed to nominal top Si-doped n^+^ region (layer 1), undoped n_0_ region (layer 2), bottom Si-doped n^+^ region (layer 3), undoped GaN buffer (layer 4), and GaN template.

**Fig. 3:**
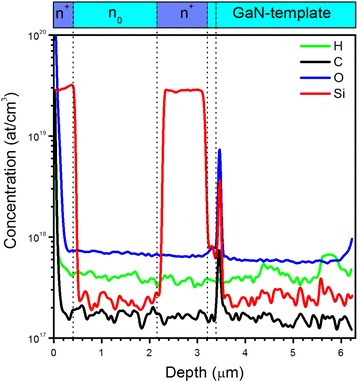
Impurity elements profiles of the investigated n^+^/n_0_/n^+^-GaN structure measured by SIMS from the sample surface

Further, in order to have the deeper insight on the impurity/doping level of the investigated samples, SIMS measurements were performed. The obtained SIMS profiles (Fig. 3) are in good correlation with the nominal thickness of GaN layers and the overall thickness of the studied multilayer structure. All examined elements (H, C, O, Si) were above the detection limit (3 to 5 × 10^16^ at/cm^3^) of SIMS technique.

Profile of intentional Si doping, in general, agrees with the nominal doping profile with the concentration of about 2.8 × 10^19^ cm^−3^ in the doped top and bottom n^+^ regions and of about 2.3 × 10^17^ cm^−3^ in the undoped n_0_ region. However, as can be seen from SIMS data, there is also thin (<50 nm)-delta layer with Si concentration of 1.1 × 10^19^ cm^−3^ between the GaN buffer and GaN template. It should be noted that Si-doped delta layer also contains higher concentrations of unintentional oxygen and carbon impurities of 2.4 × 10^19^ cm^−3^ and 1.4 × 10^18^ cm^−3^, correspondingly. This delta layer is related with homoepitaxial regrowth interface, which typically arises from the GaN template contamination with O, Si, and C impurities, absorbed from the atmosphere in the technological process of loading or at the beginning of the regrowth [25, 26].

**Fig. 4:**
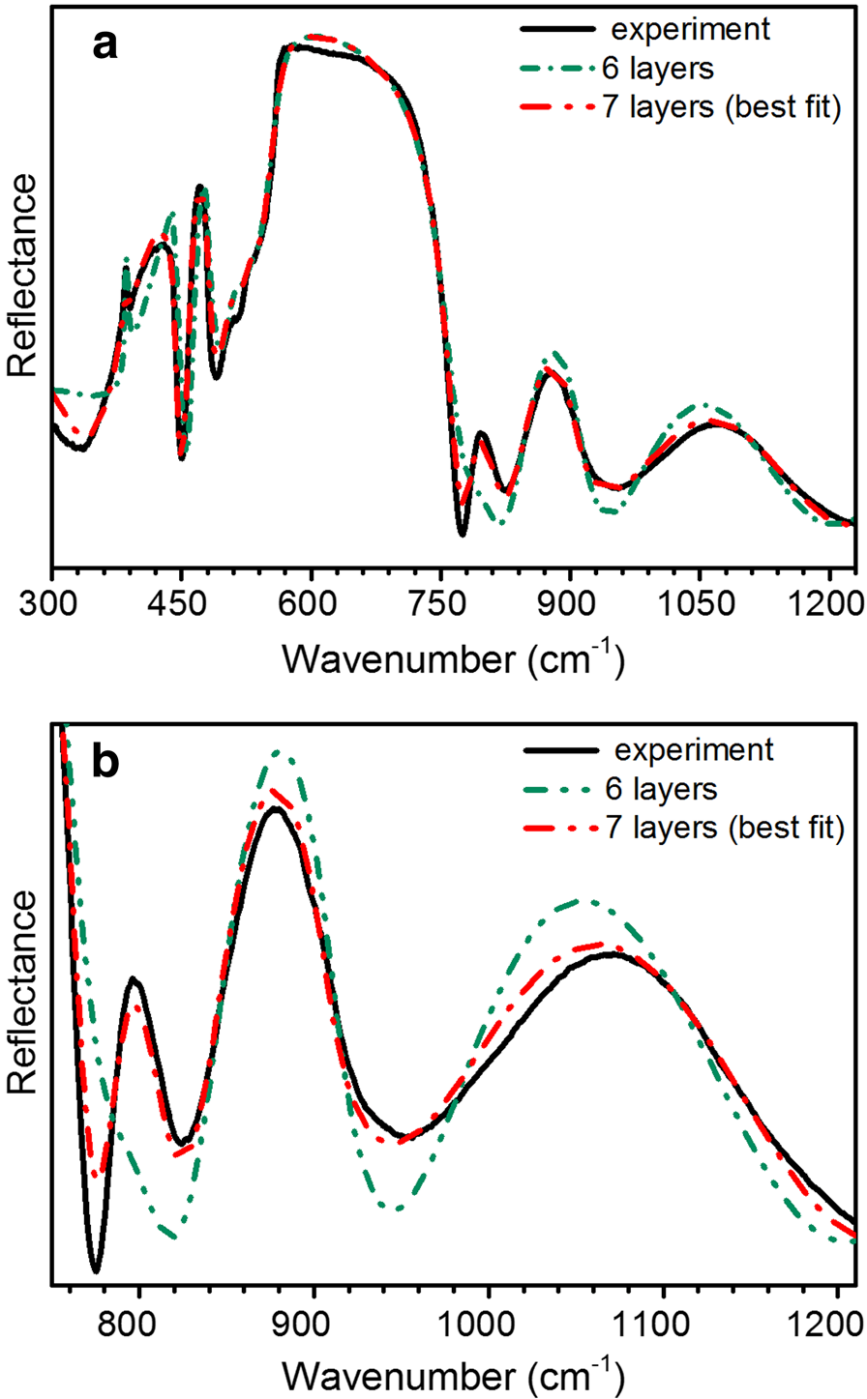
Simulations of the IR reflectance spectra with different number of layers. The experimental spectrum of the investigated n^+^/n_0_/n^+^-GaN structure is shown by *solid line*. **a** Reststrahlen region. **b** The enlarged spectra in the range above 750 cm^−1^

As discussed above, SEM cross-section and SIMS analysis give the structure of GaN layers, which differs from the nominal parameters by exciting of the additional GaN region, but with the overall thickness in agreement with the nominal one. In order to clarify the influence of additional GaN delta-layer found above on the IR reflectance spectrum of the investigated structure, the simulation of the experimental spectrum was performed by constructing models consisting of six layers, which correspond to nominal technological parameters, SEM images (Fig. 1), and seven layers according to SIMS. The calculated spectra based on the described above models are given in Fig. 4.

**Fig. 5:**
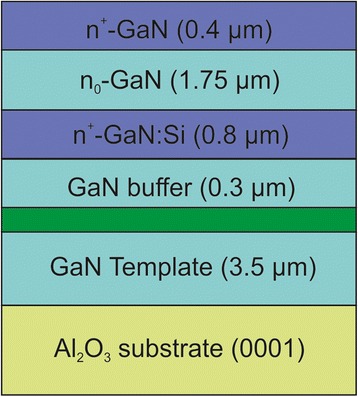
The 7-layer model used to simulate the IR reflectance spectra of the investigated n^+^/n_0_/n^+^-GaN structure. An additional layer (*green*) is thin interface layer between GaN template and the investigated GaN layers

As can be seen from Fig. 4, based on SIMS profile seven-layer model gives the best approximation of the experimental IR reflectance spectrum. Thus, further simulations and analysis are performed using this model having modified parameters, as compared to nominal technological ones (Fig. 1), and which accounts for the additional layer between the technological GaN buffer layer and GaN template (Fig. 5).

**Fig. 6:**
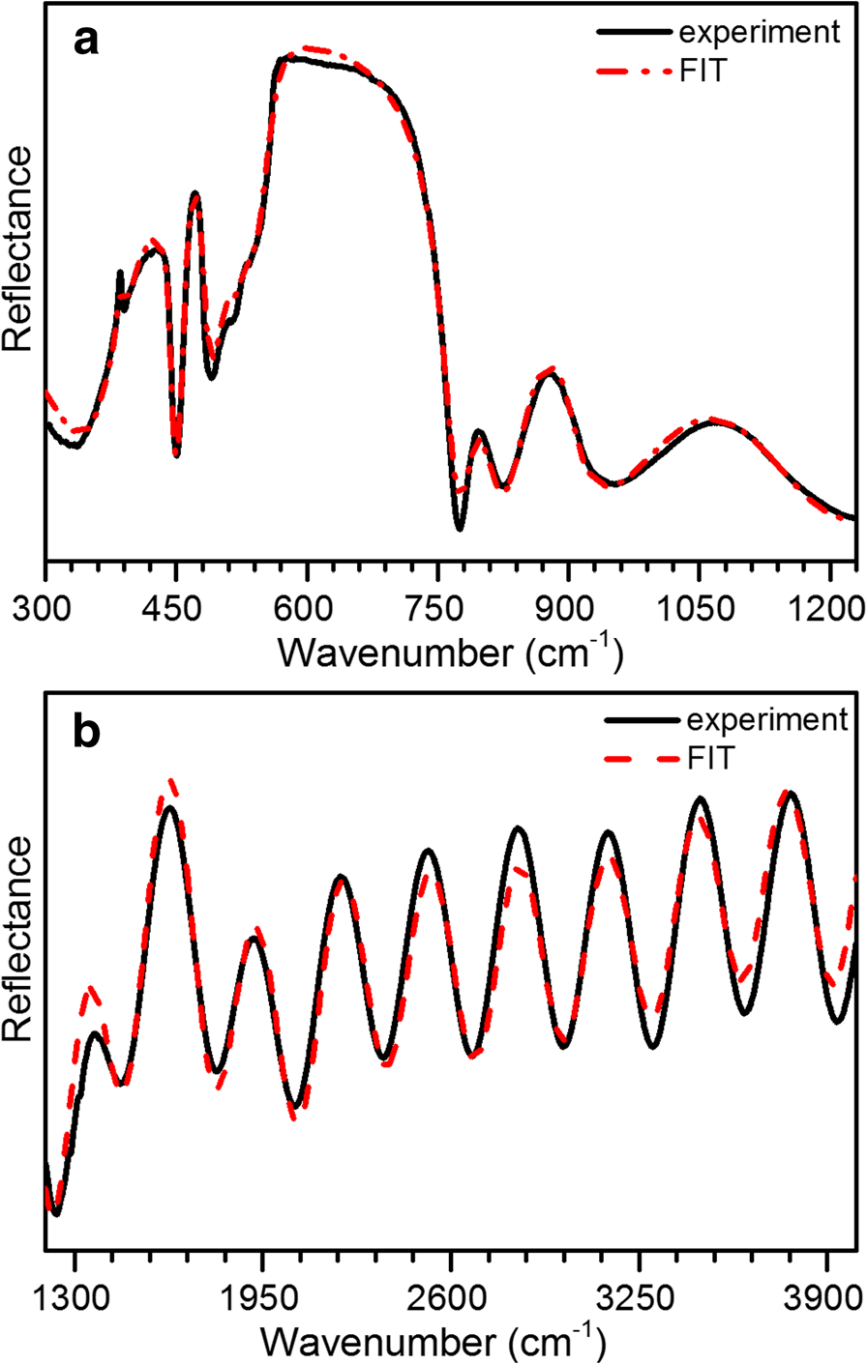
Experimental (*solid line*) and best-fit calculated (*dash-dot line*) IR reflectance spectra of the n^+^/n_0_/n^+^-GaN structure grown on GaN-template/Al_2_O_3_. **a** Reststrahlen region. **b** Interference region

Figure 6 shows experimental and fitted theoretical *s*-polarized reflectance spectra of the investigated structure at the 11° angle of incidence. The calculated spectrum is based on the model described above (Fig. 5). Dispersion of complex refractive index for the GaN layers and the sapphire substrate was determined using Eq. (5). The sapphire substrate was considered as semi-infinite, that allowed neglecting the internal reflections within the substrate and from the non-polished backside. The complicated structure observed in the reststrahlen region of the spectrum is due to a combination of the overlapping GaN and Al_2_O_3_ reststrahlen bands along with interference effects. Comparison of these data to the calculated spectra not only can provide thickness information on the various layers of the samples, but can also help to interpret the complicated structure of the reststrahlen region in terms of the contributions of the various materials.

The determination of layer thicknesses from the comparison of the reflectance data to the calculated spectrum is a two-step process [27]. First, the fringes in the transparent region above the reststrahlen bands (ω > 1200 cm^−1^) are due to interference effects on the layers of the multi-layer structure. In this way, the overall thickness of the investigated structure, which is a sum of all layers, can be estimated.

Once the stack thickness is known, the individual thicknesses of each layer can be determined by fitting the calculated spectra to interference effects in the reststrahlen region of the spectrum. Layer thicknesses were varied by taking into account the previously determined overall thickness. Under this constraint, the reflectance above 1200 cm^−1^ does not change significantly. The interference effects in the reststrahlen region can be distinguished from other features such as TO and LO vibrational modes based on the fact that the interference fringes shift in position as the layer thicknesses are varied [28].

During the approximation of the experimental spectrum in the reststrahlen region, the following model parameters were varied: damping parameters γ_LO_ and γ_TO_ for *E*_1_(LO) and *E*_1_(TO) phonon modes; plasma frequency *ω*_*p*_; plasmon damping parameter *γ*_*p*_; and layer thicknesses. It should be noted, that only *E*_1_ symmetry phonons are IR active in *s*-polarization [9]. Initial frequencies of *E*_1_(LO) and *E*_1_(TO) phonons for GaN and sapphire substrate were taken from the IR reflectance [29] and Raman scattering [6, 14] experiments. Typical values of GaN phonon frequency are ω_TO_ = 560 cm^−1^ and ω_LO_ = 740 cm^−1^. The phonon frequencies for each layer were refined in the fitting process. Obtained best-fit parameters with the error bars are given in Table 1. It should be noted that obtained in the fitting process layer thicknesses are in good agreement with the SEM data.

**Table 1:** Best fit oscillator parameters for GaN layers of the investigated structure (layers are numbered from top to bottom)

Layer no.	ω_LO_ (cm^-1^)	γ_LO_ (cm^−1^)	ω_TO_ (cm^−1^)	γ_TO_ (cm^−1^)	ω_p_ (cm^−1^)	γ_p_ (cm^−1^)	d_IR_ (μm)	d_Nominal_ (μm)	ε_∞_
1	740.2 (±0.5)	10.7 (±1.1)	561.1 (±0.8)	15.7 (±0.9)	507.8 (±1.2)	350.5 (±1.0)	0.47 (±0.02)	0.401	5.25 (±0.07)
2	740.7 (±0.8)	12.8 (±0.9)	560.8 (±0.2)	14.4 (±0.5)	55.7 (±0.5)	155.7 (±0.5)	1.73 (±0.06)	1.752	5.01 (±0.03)
3	740.4 (±0.3)	11.4 (±1.0)	562.3 (±0.4)	6.83 (±0.5)	537.1 (±0.9)	390 (±0.7)	0.8 (±0.02)	0.82	5.35 (±0.08)
4	740.1 (±0.7)	6.22 (±0.7)	560.7 (±0.1)	17.8 (±0.5)	132.3 (±0.5)	249.1 (±0.5)	0.27 (±0.03)	0.31	5.35 (±0.1)
5	742.1 (±0.2)	8.68 (±0.8)	560.4 (±0.7)	18.4 (±0.5)	436.1 (±0.7)	383.3 (±0.5)	0.01^a^ (±0.001)	-	5.3 (±0.09)
Template	741.5 (±1.0)	13.14 (±1.3)	560.2 (±0.3)	7.16 (±0.1)	51.39 (±0.8)	180 (±0.9)	3.48 (±0.05)	3.51	4.99 (±0.07)

^a^Thickness of interface layer was not determined from SEM data

**Fig. 7:**
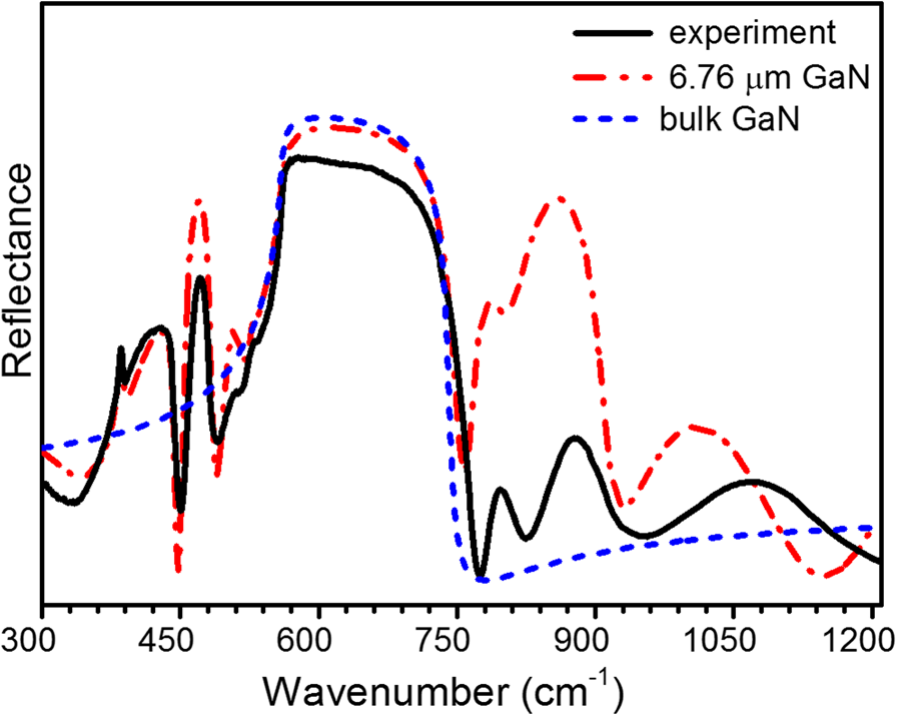
Experimental (*solid line*) IR reflectance spectra of the investigated n^+^/n_0_/n^+^-GaN structure and calculated reflectance spectra of 6.78-μm-thick GaN layer on sapphire (*dash-dot line*) and bulk GaN (*dash line*)

Referring to Fig. 6a, the reflectance peak at ~450 cm^−1^ can be attributed to the sapphire substrate. The features observed in the range of 500–740 cm^−1^ are due to a combination of overlapping features from GaN layers and sapphire reststrahlen bands. For the deeper analysis, the IR reflectance spectra of bulk GaN and 6.78-μm-thick GaN layer on sapphire, with the thickness of GaN corresponding to the overall thickness of the investigated structure, were simulated in the reststrahlen band region (Fig. 7). As can be seen from Fig. 7, the reflectance spectra of 6.78-thick GaN layer on sapphire and bulk GaN in the range of 500–740 cm^−1^ are similar to the experimental spectrum. The small feature at ~511 cm^−1^ is associated with the sapphire substrate. It should be mentioned that at ~736 cm^−1^, there is a weak dip that corresponds to *A*_1_(LO) mode of GaN template. According to the selection rules, *A*_1_(LO) mode is forbidden in *s*-polarized IR spectra [9]. The possible reason for the registration of this forbidden mode could be a polarization leakage due to the aperture of the reflectance accessory as well as microinhomogeneities of GaN crystal structure. Specifically, this can be caused by inclination of the *c*-axis of the column-like wurtzite structure of GaN from the direction perpendicular to the film’s growth plane. This mode was not taken into account in our modeling because of its weak impact on the resulting spectrum. The features in the range of 750–1200 cm^−1^ are due to overlapping GaN:Si and sapphire reststrahlen bands and interface effects. The drop at ~775 cm^−1^ is related to interface effect on the edge of the reststrahlen band of GaN layers and sapphire. The broad dip at ~825 cm^−1^ is associated with overlapping of the high-frequency branch of the plasmon-LO-phonon coupled mode (LPP^+^) of the n^+^ layers.

**Fig. 8:**
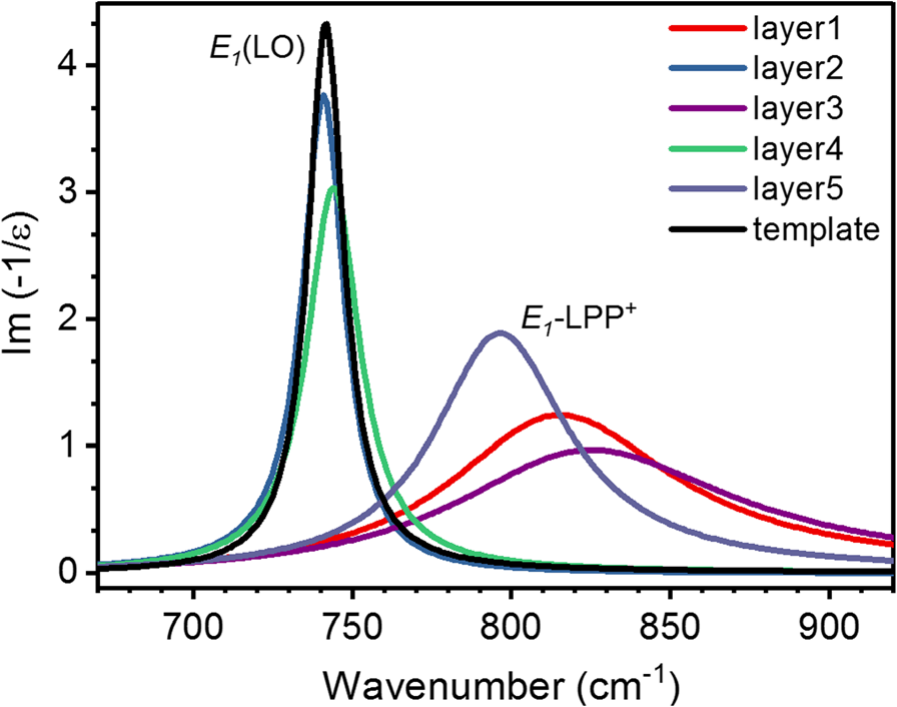
Calculated imaginary part of dielectric function obtained for each analyzed GaN layer from the best-fit data analysis

Figure 8 shows the calculated imaginary parts of the energy loss function for each layer according to oscillator parameters given in Table 1 for estimation of *E*_1_-LOPC modes. As can be seen, the high-frequency branch of the LOPC modes (LPP^+^) at carrier concentrations lower than 10^17^ cm^−3^ (n_0_ layer and template) almost coincide with *E*_1_(LO) phonon mode. The increase in carrier concentration in the range of 2 × 10^17^–3 × 10^18^ cm^−3^ (Fig. 5) leads to significant high-frequency shift and broadening of the LPP^+^ branch, which indicates the increase in interaction between LO phonon and plasmon and the decrease in mobility of charge carriers. This behavior of LPP^+^ branch agrees well with the experimental data on IR reflectance of Si-doped GaN films grown on sapphire by Z.F. Li et al. [30], and Raman measurement in bulk GaN [10] and epitaxial layers [31]. It should be noted that the low-frequency LPP^−^ branch of the LOPC cannot be reliably defined in our case, as *s*-polarized IR reflectance spectra were not measured in low-frequency range below 300 cm^−1^.

Values of carrier concentration and mobility listed in Table 2 were calculated using Eqs. (8) and (9) with electron effective mass m* of 0.2 m_0_ [32]. It can be seen, that calculated carrier concentration profile is similar to the Si impurity concentration profile obtained by SIMS measurements (Fig. 3), but with the order of magnitude lower carrier concentrations as compared to concentration Si impurity. Such discrepancy in concentrations of carriers and doping impurities was observed earlier by M. Bockowski et al. [33], and was related to compensation effects by acceptor states (likely by gallium vacancies), formation energy of which lowers with increasing n-type doping [34]. It should be mentioned, that carrier concentration for the n^+^ layers in the order of ~10^18^ cm^−3^ is in good agreement with the results of our Raman studies of similar GaN structures based on analysis of LOPC modes [6]. Obtained decrease of carrier mobility μ with carrier concentration is also in good agreement with Hall experiments in GaN [35] and theoretical modeling [36].

**Table 2:** Optically determined values of carrier concentration and mobility for each analyzed GaN layer of the investigated n^+^/n_0_/n^+^-GaN structure

Layer no.	*N* × 10^17^ (cm^−1^)	μ (cm^2^V^−1^s^−1^)
1	28.8 (±0.13)	133.3 (±1.20)
2	0.37 (±0.01)	300.0 (±0.90)
3	34.4 (±0.12)	119.7 (±0.75)
4	2.04 (±0.03)	187.4 (±0.67)
5	22.20 (±0.07)	121.9 (±0.52)
Template	0.31 (±0.01)	259.4 (±1.27)

The values of high-frequency dielectric permittivity ε_∞_ were found to be in the range of 4.99–5.35 (Table 1). The increase in ε_∞_ for the doped n^+^ layers as compared to n_0_ layers can be related to the red shift of the α-GaN band gap [37]. It should be noted that values of ε_∞_ can be determined with relatively small error only for low-conductive films. Accuracy in the determination of ε_∞_ decrease with carrier concentration, which is related to the fact that the ε_∞_ parameter accounts for “the high-frequency” limit when the dielectric model function is extrapolated to shorter wavenumbers than those studied here [11]. The wide spectral range of 300–4000 cm^−1^ was analyzed in order to decrease the error in the determination of ε_∞_ and other parameters involved in modeling the IR reflectance spectra of n^+^ layers.

## Conclusions

IR reflectance spectra of the multilayer structure consisting of GaN layers grown on a sapphire substrate and doped with different concentrations of Si impurity were measured and analyzed in details. Analysis of the investigated structure by SEM of photo-etched cross-section showed good correlation with the technological parameters of the GaN layers. SIMS analysis also revealed the presence of thin delta layer near the GaN buffer/GaN-template interface with higher content of Si and O impurities, which is related to homoepitaxial regrowth interface. Modeling of IR reflectance spectrum of the studied multilayer structure by including into analysis the additional layer made it possible to obtain the best fitting of the experimental spectrum. Obtained thicknesses of GaN layers are in good agreement with the SEM and SIMS data. Calculated from the spectral dependence of dielectric permittivity LOPC modes for each GaN layer showed high-frequency shift and broadening of LPP^+^ branch with the increase in carrier concentration. Concentration and mobility of charge carrier for each GaN layer were calculated from the plasmon frequency and damping parameter. Obtained carrier concentration profile is similar to those obtained by SIMS, but with values of carrier concentration one order of magnitude less than the concentration of Si doping impurity, which can be attributed to compensation effects by the defect acceptor states. Thus, it is demonstrated that IR reflectance spectroscopy and 2 × 2 transfer matrix method can be successfully used for analysis of epitaxial multilayer GaN structures with non-uniform doping profiles, and allow for the determination of the fundamental electron and phonon parameters of each GaN layer.

## Abbreviations

IR: Infrared
FTIR: Fourier transform infrared spectroscopy
SEM: Scanning electron microscopy
SIMS: Secondary ion mass spectrometry
LOPC: Longitudinal optical phonon*–*plasmon coupled
