# Erratum to: Infrared Reflectance Analysis of Epitaxial n-Type Doped GaN Layers Grown on Sapphire

**DOI:** 10.1186/s11671-017-2227-1

**Published:** 2017-08-21

**Authors:** Bogdan I. Tsykaniuk, Andrii S. Nikolenko, Viktor V. Strelchuk, Viktor M. Naseka, Yuriy I. Mazur, Morgan E. Ware, Eric A. DeCuir, Bogdan Sadovyi, Jan L. Weyher, Rafal Jakiela, Gregory J. Salamo, Alexander E. Belyaev

**Affiliations:** 1 V. Lashkaryov Institute of Semiconductor Physics, National Academy of Sciences of Ukraine, Pr. Nauky 41, Kiev, 03680 Ukraine; 2 Institute for Nanoscience and Engineering, University of Arkansas, West Dickson 731, Fayetteville, AR 72701 USA; 3 Institute of High Pressure Physics, Polish Academy of Sciences, Sokolowska str. 29/37, 01-142 Warsaw, Poland; 4 Institute of Physics, Polish Academy of Sciences, Al. Lotnikow 32/46, PL-02-668 Warsaw, Poland

## Erratum

In the original publication [1] was equation 2 not correctly displayed in the online PDF.

Furthermore reference 1 and 2 were incorrect. The correct versions can found below:

Incorrect equation in the online PDF:

2 $$ {T}_{l/\left( l+1\right)}^{s/ p}=\frac{1}{t_{l/\left( l+1\right)}^{s/ p}}\left( \exp \left( i{\delta}_l^{s/ p}\right)-{r}_{l+1, l}^{s/ p} \exp \left( i{\delta}_l^{s/ p}\right){r}_{l, l+1}^{s/ p} \exp \left(- i{\delta}_l^{s/ p}\right) \exp \left(- i{\delta}_l^{s/ p}\right)\right), $$

Correct equation in the online article:

2 $$ {T}_{l/\left( l+1\right)}^{s/ p}=\frac{1}{t_{l/\left( l+1\right)}^{s/ p}}\left(\begin{array}{cc}\hfill \exp \left( i{\delta}_l^{s/ p}\right)\hfill & \hfill -{r}_{l+1, l}^{s/ p} \exp \left( i{\delta}_l^{s/ p}\right)\hfill \\ {}\hfill {r}_{l, l+1}^{s/ p} \exp \left(- i{\delta}_l^{s/ p}\right)\hfill & \hfill \exp \left(- i{\delta}_l^{s/ p}\right)\hfill \end{array}\right), $$

Incorrect reference 1

1. Manasreh MO, Weaver BD (2001) Local vibrational modes of carbonhydrogen complexes in proton irradiated AlGaN. Mater Res 692:403–409

Should be exchanged with this one:

- Davis RF (1991) III-V Nitrides for Electronic and Optoelectronic Applications. Proc IEEE 79: 702-712

Incorrect reference 2

2. Sun WH, Chen KM, Yang ZJ, Li J, Tong YZ, Jin SX, Zhang GY, Zhang QL, Qin GG (1999) Using Fourier transform infrared grazing incidence reflectivity to study local vibrational modes in GaN. J Appl Phys 85:6430–6433

Should be exchanged with this one:

- Xing H, Keller S, Wu Y-F, et al (2001) Gallium nitride based transistors. J Phys Condens Matter 7139: 7139-7157

The equation in the online article has been updated to rectify this error.
